# Blockers of the SARS-CoV-2 3a Channel Identified by Targeted Drug Repurposing

**DOI:** 10.3390/v13030532

**Published:** 2021-03-23

**Authors:** Prabhat Pratap Singh Tomar, Miriam Krugliak, Isaiah T. Arkin

**Affiliations:** Department of Biological Chemistry, The Alexander Silberman Institute of Life Sciences, The Hebrew University of Jerusalem, Edmond J. Safra Campus Givat-Ram, Jerusalem 91904, Israel; ppstdbt@gmail.com (P.P.S.T.); miriamkru@savion.huji.ac.il (M.K.)

**Keywords:** viral channels, bacterial assays, channel blockers, antiviral drugs

## Abstract

The etiological agent of the COVID-19 pandemic is SARS-CoV-2. As a member of the Coronaviridae, the enveloped pathogen has several membrane proteins, of which two, E and 3a, were suggested to function as ion channels. In an effort to increase our treatment options, alongside providing new research tools, we have sought to inhibit the 3a channel by targeted drug repurposing. To that end, using three bacteria-based assays, we screened a library of 2839 approved-for-human-use drugs and identified the following potential channel-blockers: Capreomycin, Pentamidine, Spectinomycin, Kasugamycin, Plerixafor, Flumatinib, Litronesib, Darapladib, Floxuridine and Fludarabine. The stage is now set for examining the activity of these compounds in detailed electrophysiological studies and their impact on the whole virus with appropriate biosafety measures.

## 1. Introduction

The COVID-19 pandemic has engulfed the world in a manner that few, if any, other diseases have. Within a year of its emergence in December of 2019, roughly 100 million people were infected by the virus leading to more than two million deaths [1]. Its repercussions on the economy, particularly on emerging markets [2], and its impact on our social fabric [3] have been commensurately sizable.

The disease’s influence has spurred considerable research efforts. The pandemic’s etiological agent was identified rapidly as a new member of the Coronaviridae [4,5], which is very similar to the Severe Acute Respiratory Syndrome (SARS) pandemic’s causative agent [6,7]. Accordingly, the virus was therefore named SARS-CoV-2 [8].

Efforts to halt viral infectivity have proceeded in several avenues. Vaccine development utilizing different technologies has advanced at an unprecedented pace, with multiple candidates receiving regulatory approvals in less than a year [9,10,11,12,13]. Convalescent plasma therapy has also been employed for COVID-19 treatment with some success [14,15,16]. Finally, drug therapy against COVID-19 has also been extensively examined [17]. Agents that abate the onslaught of the immune system’s cytokine storm have proven to represent a promising approach [18,19]. However, with the possible exception of remdesivir [20] (efficacy contention notwithstanding [21,22]), drugs that target the virus directly (i.e., antivirals) have been less forthcoming.

Considering the above, we have sought to identify new antiviral agents by directly targeting an ion channel in the virus. Our reasoning stems from the fact that channels as a family are excellent targets for pharmaceutical point intervention [23]. Consequently, channels, present in numerous viruses [24,25], have also been suggested to serve as attractive drug targets [26]. Yet, so far, the only approved compounds that inhibit a viral channel are the anti-flu aminoadamantanes [27,28,29]. Amantadine and rimantadine target the virus’s M2 protein [30] by blocking its H^+^ channel activity [31], albeit with wide-spread resistance [32].

As a coronavirus, SARS-CoV-2 is an enveloped pathogen with several proteins in its membrane that have been purported to exhibit channel activity: E, 3a, 4a and 8a [33,34]. Moreover, such channels may play a role in the viral infectivity cycle [35]. Among these proteins, the channel activity of E and 3a have been the most characterized both in SARS-CoV-2 [35] (although detailed electrophysiological data are missing for 3a [34]), and other Coronaviridae members [36,37,38,39,40,41,42,43,44,45,46,47,48,49,50,51]. In particular, we note detailed structural analyses of the E [52] and 3a [53] proteins.

Since we have already undertaken fruitful screening efforts against the E protein [35,54], we have decided to target 3a in the current study. Finally, to minimize the chemical search space and potentially expedite future regulatory steps, we focused on compounds that have been approved for human use. Drug repurposing as such has proven to be a valuable avenue towards drug discovery [52].

## 2. Results

The goal of our study was to identify blockers of the 3a channel. To do so, we made use of constructs in which the viral channel is heterologously expressed in bacteria, and as a consequence, alters their phenotype. Subsequently, blockers may be identified if they reverse the channel-induced characteristics.

In all bacterial assays SARS-CoV-2 3a was expressed as a chimera, fused to the carboxy terminus of the maltose binding protein (MBP Fusion and Purification System, New England BioLabs, Ipswich, MA). This system ensures targeting of the protein to the inner bacterial membrane. While this membrane may not be identical to the eukaryotic Golgi-ER compartment where 3a is thought to reside [55,56], it was used to express and examine many other viral ion channels successfully [35,53,55,57,58].

### 2.1. 3a Channel Activity

Three bacteria-based assays were employed to examine channel activity and identify inhibitors thereof. Below, we describe each assay in detail and demonstrate the experiential results of the protein accordingly.

#### 2.1.1. Negative Assay

The first assay that we employed involved the expression of the viral channel at increasing levels in “ordinary” *Escherichia coli*. At a certain viroporin concentration, growth retardation will be observed due to excessive membrane permeabilization that hampers bacterial bioenergetics [57]. This test is therefore termed a negative assay due to the detrimental impact that the protein has on the bacteria. Finally, we recognize this assay’s inherent deficiency is its inability to detect efficacious compounds if they are toxic to bacteria or incapable of penetrating the bacterial outer membrane.

**Figure 1:**
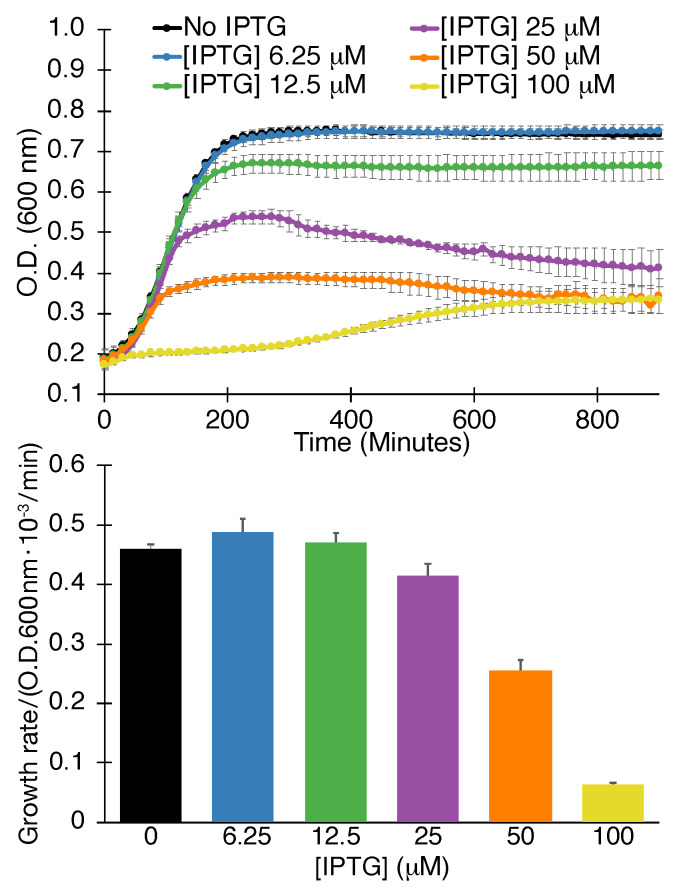
Negative assay. Growth curves (**top**) and maximal growth rates (**bottom**) of bacteria as a function of SARS-CoV 3a protein expression, governed by the level of the inducer β-d-1-thiogalactopyranoside (IPTG), as indicated. The analyses were performed in triplicates with indicated standard deviations.

Results shown in Figure 1 demonstrate that SARS-CoV-2 3a scores positively in the negative assay. Explicitly, increasing the concentration of the expression inducer (IPTG) results in lowering the growth of bacteria. Moreover, both the maximal growth rate is decreased, as well as the final culture density.

We recognize that numerous factors may result in lower bacterial growth when expressing a heterologous protein. However, such spurious results may be discounted with the following two assays described below, as well as identifying blockers that increase growth in the current (negative) assay.

#### 2.1.2. Positive Assay

The second assay we used entailed expression of the viral channel at lower levels in K^+^-uptake deficient bacteria [56]. Such bacteria are incapable of growing in regular media unless they are supplemented by potassium, or when they express a channel capable of K^+^ transport. In this instance, the viral channel impacts the bacteria favorably and the assay is therefore termed positive assay. Note, that at high induction levels, while alleviating the K^+^ shortage in the bacteria, the channel impacts the bacteria negatively due to excessive membrane permeabilization akin to the negative assay discussed above.

**Figure 2:**
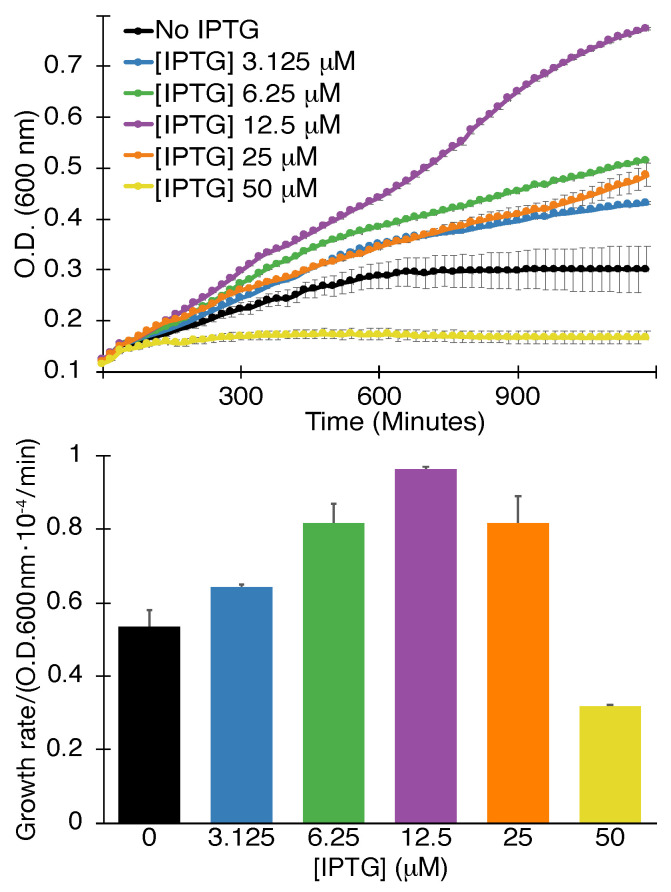
Positive assay. Growth curves (**top**) and maximal growth rates (**bottom**) of K^+^-uptake deficient bacteria as a function of SARS-CoV 3a protein expression, governed by the level of the inducer β-d-1-thiogalactopyranoside (IPTG), as indicated. The analyses were performed in triplicates with indicated standard deviations.

The results shown in Figure 2 indicate that SARS-CoV-2 3a passes the positive assay as well. Specifically, increasing the inducer concentrations up to 12.5 μM enhances bacterial growth rate and final density accordingly. Inducer concentrations larger than 12.5 μM are detrimental to growth as expected due to deleterious membrane permeabilization.

The positive assay’s results can dispel the impact of any spurious factors in the negative assay effectively. In other words, it is difficult to imagine that non-specific factors cause the 3a protein to retard the growth of regular bacteria on one hand while being beneficial to the growth of K^+^-uptake deficient bacteria on the other hand.

#### 2.1.3. pH Assay

The final assay that examines channel activity is based on the impact that a channel has on the cytoplasmic pH of bacteria. When a concentrated acid is injected into the media, the cytoplasmic pH will drop if the bacteria express a channel capable of H^+^ transport. Subsequently, a change in cytoplasmic pH can be detected by monitoring the fluorescence of a chromosomally-expressed, pH-sensitive GFP [59].

**Figure 3:**
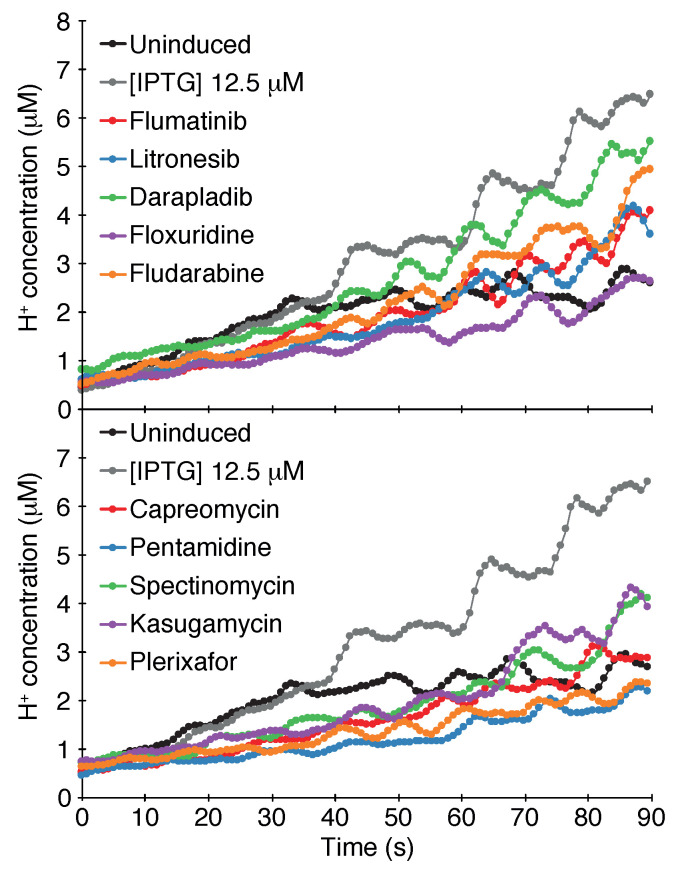
pH assay. Fluorescence of bacteria that harbor pHluorin, a pH sensitive GFP [59], was examined as a function of SARS-CoV-2 3a protein expression and different chemicals as indicated. The drugs were added at 50 μM concentration. The H^+^ concentration was determined as detailed in the Materials and Methods section. The analyses were performed in quadruplicates.

Results shown in Figure 3 indicate that the SARS-CoV-2 3a channel passes the pH assay as well. Induction of the protein with 12.5 μM β-d-1-thiogalactopyranoside causes an appreciable increase in the cytoplasmic H^+^ concentration in comparison to bacteria in which the channel is uninduced.

### 2.2. Screening Results

Following confirmation of the 3a protein channel activity in all three bacterial assays, we set forth to screen a library of 2839 approved-for-human-use drugs (MedChem Express, Monmouth Junction, NJ, USA). Our screening strategy (at 100 μM concentration) employed a three-tier system as follows: We started by screening each of the 2839 chemicals using the negative assay. Following, any compound that was successful in reviving bacterial growth was retested in the negative assay in duplicates. Subsequently, compounds that repetitively exhibited the ability to enhance bacterial growth in the negative assay were tested in the positive assay in duplicates. Any compound that passed both the negative and positive assays was designated as a hit and was subjected to a Dose-response analysis in both tests. Finally, the hits were also analyzed using the pH assay for final confirmation.

**Figure 4:**
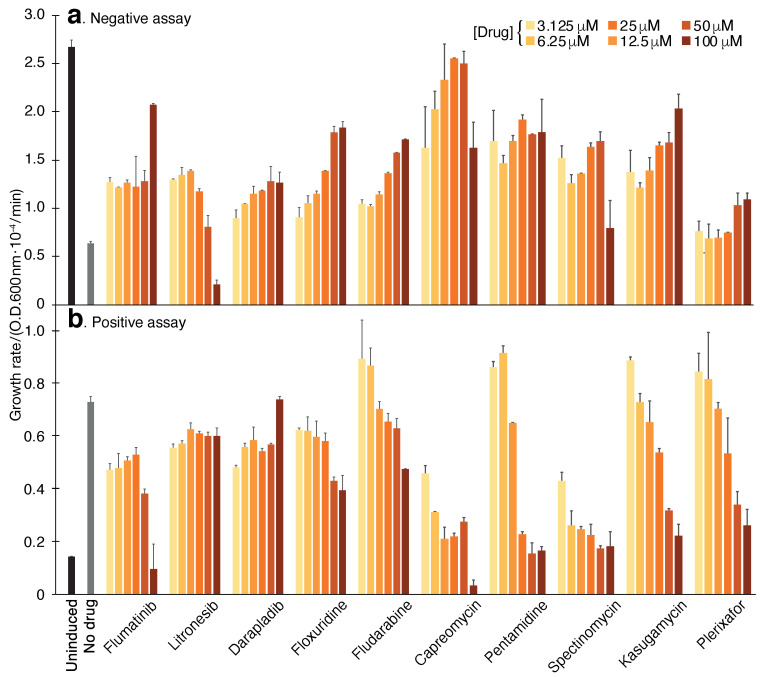
Compound screening results using the negative and positive assays. (**a**). Negative assay in which SARS-CoV-2 3a protein is expressed at an elevated level (induction with 100 μM β-d-1-thiogalactopyranoside) and is therefore deleterious to DH10B bacteria. In this instance, inhibitory drugs enhance bacterial growth. (**b**). Positive assay in which SARS-CoV-2 3a protein is expressed at a low level (12.5 μM β-d-1-thiogalactopyranoside) in K^+^-uptake deficient bacteria (LB650). In this instance, inhibitory drugs reduce bacterial growth. The results in both panels may be compared to those obtained without any drug (gray) or when the channel is uninduced (black). The color scale indicates the different concentrations of the chemicals, which were performed in duplicates.

The results of the screening identified ten compounds that scored positively in all of the assays: Capreomycin, Pentamidine, Spectinomycin, Kasugamycin, Plerixafor, Flumatinib, Litronesib, Darapladib, Floxuridine and Fludarabine. The numerical results of the negative and positive tests at a concentration of 50 μM are shown in Table 1. Dose-response analyses of each compound in the negative and positive assays are shown in Figure 4. Finally, the results of the pH assay are shown in Figure 3.

## 3. Discussion

Compounds capable of modulating ion channel activity are a very successful drug class, second only to agents that target G protein-coupled receptors. Amongst numerous examples, one might list: dihydropyridines, used to treat hypertension due to their ability to block L-type calcium channels; sulfonylureas and metiglinides used for the treatment of diabetes due to their ability to block ATP-dependent K^+^ channels; and local anesthetics such as lidocaine that block Na^+^ channels [23].

Since many viruses were shown to contain ion channels [24,25], by inference, blocking such proteins may represent a promising avenue towards antiviral drug development [26]. However, only one class of compounds, the anti-flu aminoadamantanes [27,28,29], are currently approved as antiviral drugs. Hence, one might consider viral ion channels an underexploited opportunity for pharmaceutical point intervention.

Considering the emerging need for treatment options against the COVID-19 pandemic, we set forth to identify blockers against one of the channels found in the disease’s etiological agent: the 3a protein. This effort complements our previous and concurrent efforts to identify blockers against the E protein, another one of SARS-CoV-2’s channels [35,54].

Our strategy employed three independent bacteria-based assays for the following reasons: (i) analyses of the channel are conducted in a biological membrane, albeit of bacterial origin; (ii) changes in the sequence (i.e., mutations) are easy to implement rapidly; (iii) the assay is amenable to screening thousands of chemicals per month in an academic setting; (iv) bacterial tolerance enables analysis of a wide range of chemical concentrations; (v) these assays provide inherent controls to one another, thereby minimizing false positive hits.

Our results have yielded 10 chemicals that scored positively in all three assays (Table 1). We recognize that it is possible to speculate that the impact of the drugs in each of the individual assays may be attributed to indirect or non-specific factors, i.e., not by inhibiting the 3a protein directly. However, the fact that each of the hits scored positively in three independent tests (two of which are reciprocally related), reduces this possibility appreciably. Figure 4 is particularly illustrative of this fact, whereby a mirror image is seen in the dose-response curves of individual chemicals.

For example, pentamidine diminishes the deleterious impact that the 3a protein has on the bacteria by enhancing the growth rate by 177%. Reciprocally, the same chemical lowers the growth rate of K^+^-uptake deficient bacteria that rely on the 3a protein to thrive in low K^+^ media by 83%. In the pH assay, it reduces the acidification of the bacterial cytoplasm which is caused by the 3a protein. Finally, it is important when comparing the individual assays’ results to one another to remember that they are not entirely quantitative. Therefore, one should not expect a strict correlation between the individual assays.

Due to the medical importance of COVID-19, it is not surprising that there have been numerous repurposing studies aimed at inhibiting different proteins in the virus. However, while most are in silico analyses, the experimental study of Riva and colleagues stands out [60]. The authors describe screening ca. 12,000 repurposed compounds directly on viruses replicating on tissue culture cells at 5 μM concentration. None of the hits that our screen retrieved were identified by this large repurposing study, perhaps due to the different stringencies in the two studies. The current study screened every chemical at 100 μM, whereas Riva and coworkers employed 5 μM. Screening at 100 μM stemmed from our desire to cast a wide net, which is possible in the more tolerant bacterial system. Molecules that emerge from our bacterial screen with lower affinities, may still be beneficial, by serving as a starting point for further chemical exploration. Moreover, drugs that block the 3a channel in low-affinity may interact synergistically with inhibitors of other targets in the virus.

Future electrophysiological studies will be needed to assess the detailed inhibitory mechanisms of each chemical. In addition, in vitro and in vivo studies will be required to establish the anti-viral activity of the different blockers considering the intracellular localization of the protein and the ability of individual hits to access it [55,56].

## 4. Materials and Methods

### 4.1. Channel Assays

Three bacteria-based assays were performed as described previously when analyzing the E protein from SARS-CoV-2 [35,55].

#### 4.1.1. Negative Assay

DH10B bacterial cultures were grown overnight, diluted 500 fold and grown until their growth density reached an O.D._600_ of 0.2. Following, 50 μL of culture were placed in 96-well flat-bottomed plates that contained 50 μL of the different treatments. Induction was subsequently achieved by adding β-d-1-thiogalactopyranoside at 100 μM. d-glucose was added to a final concentration of 1%. A multi-plate incubator (Tecan Group, Männedorf, Switzerland) was used to incubate the plates for 16 h at 37 °C at a constant, high shaking rate. Bacterial growth was monitored by measuring O.D._600_ every 15 min on an Infinite 200 plate reader (Tecan Group). Duplicates, or triplicates were conducted for every measurement.

#### 4.1.2. Positive Assay

The positive assay was conducted similarly to the negative assay except for the following changes: (i) The bacterial strain was LB650 which is K^+^-uptake deficient [56]. (ii) Protein induction was achieved by adding 12.5 μM of β-d-1-thiogalactopyranoside. (iii) The bacteria were grown overnight and diluted in LBK media (LB in which K^+^ was used to replace Na${}^{+}$). Thereafter, the growth medium was replaced with LB that was supplemented with 5 mM KCl.

#### 4.1.3. pH Assay

The pH assay made use of bacteria that express a chromosomal copy of a pH-sensitive GFP [61,62]. Bacterial cultures were grown overnight and subsequently diluted 1:500 in LB media. Growth was then allowed to continue until the O.D._600_ reached a value of 0.6–0.8. Protein expression was achieved by adding β-d-1-thiogalactopyranoside to a final concentration of 50 μM. Following one hour of induction, the O.D._600_ of the culture was measured and thereafter the bacteria were pelleted at 3500 g for 10 min. The bacteria were then resuspended in McIlvaine buffer (200 mM Na${}_{2}$HPO${}_{4}$, 0.9% NaCl adjusted to pH 7.6 with 0.1 M citric acid, 0.9% Nacl) to an O.D._600_ of 0.25. Then, 200 μL of bacterial suspension were transferred alongside 30 μL of McIlvaine buffer to a 96 well plate. As a control, the plate included a row with only the assay buffer and cultures without induction. Fluorescence measurements were undertaken by an Infinite F200 pro microplate reader (Tecan Group, Männedorf, Switzerland).

The experiment was initiated by adding to the bacteria, 70 μL of 300 mM citric acid with 0.9% NaCl. The fluorescence (emission at 520 nm) of each well was measured by alternating the excitation between 390 nm and 466 nm for 90 s. The proton concentration was then calculated from the ratio between the two differently excited emissions according to [61,62].

### 4.2. Chemical Screening

The chemical library was purchased from MedChem Express (HY-L035, Monmouth Junction, NJ, USA). At the time, the library contained 2839 repurposed drugs, noting that the number of chemicals changes with time. Each chemical was tested at a final concentration of 100 μM. The final concentration of dimethyl sulfoxide was 2%. All manipulations and growths were conducted on a robotic system (EVO 75 Tecan, Männedorf, Switzerland).

For each growth test, two metrics were measured: maximal growth rate and final bacterial density. However, in practice, visual inspection was far superior in identifying individual hits due to spurious factors that may influence the aforementioned metrics, such as compound absorbance, solubility, etc.

The screening proceeded in three stages. Initially, we screened all compounds in the negative assay. Each plate had two controls: the positive control were bacteria without β-d-1-thiogalactopyranoside, i.e., without channel induction. Blank DMSO addition served as a negative control. Subsequently, bacteria that experienced growth enhancement beyond an empirical threshold were reexamined in triplicate. Every compound that passed this assay was then examined in the positive assay in triplicate. Finally, compounds that passed both the positive and negative assays were subjected to a dose-response analysis in duplicate and the pH assay in quadruplicate.

## Figures and Tables

**Table 1:** Impact of drugs in the different assays. In the negative and positive assays, the values represent the growth enhancement or retardation relative to untreated bacteria, respectively. In the pH assay, the values represent the reduction in H^+^ concentration change relative to untreated bacteria. All compounds were assessed at 50 μM concentration.

Hit	Negative Assay	Positive Assay	pH Assay
Capreomycin	+292%	−70%	−59%
Pentamidine	+177%	−83%	−74%
Spectinomycin	+166%	−81%	−53%
Kasugamycin	+163%	−65%	−48%
Plerixafor	+62%	−63%	−74%
Flumatinib	+101%	−47%	−47%
Litronesib	+27%	−17%	−48%
Darapladib	+101%	−22%	−24%
Floxuridine	+180%	−41%	−68%
Fludarabine	+147%	−13%	−37%

## Data Availability

Data sharing not applicable.
